# Supplementary material for: Acupuncture Therapy for Military Veterans Suffering from Posttraumatic Stress Disorder and Related Symptoms: A Scoping Review of Clinical Studies
Source: Healthcare (Basel). 2023 Nov 14;11(22):2957. doi: 10.3390/healthcare11222957 (PMC10671227; doi:10.3390/healthcare11222957)
Supplement: Supplementary file 1 [file healthcare-11-02957-s001.zip › Certificate_of_editing-PESUY_8_4_injczkqacw.pdf]

editage

Editing Certificate

This document certifies that the manuscript listed below has been edited to ensure language and grammar accuracy and is error free in these aspects. The logical presentation of ideas and the structure of the paper were also checked during the editing process. The edit was performed by professional editors at Editage, a brand of Cactus Communications. The author's core research ideas were not altered in any way during the editing process. The quality of the edit has been guaranteed, with the assumption that our suggested changes have been accepted and the text has not been further altered without the knowledge of our editors.

MANUSCRIPT TITLE

Acupuncture therapy for military veterans suffering from posttraumatic stress disorder and related symptoms: A scoping review of clinical studies

AUTHORS

Hui-Yong Kwak, Jungtae Leem, Hye-Bin Seung, Chan-Young Kwon, Hye-Seon Jeong, Sang-Ho Kim

ISSUED ON

November 09, 2023

JOB CODE

PESUY_8_4

THE EDITAGE Promise QUALITY GUARANTEE

Prabh Grewal
Senior Vice President - Editage

editage helping you get published

Since 2002, Editage has helped over 430,000 authors publish around 1.2 million research papers in scholarly journals across over 1000 disciplines through editorial, translation, transcription, and publication support services. Editage is a brand of Cactus Communications (cactusglobal.com), a science communication and technology company.

CACTUS

GLOBAL :
+1(833) 979-0061 | request@editage.com

KOREA :
02-3478-4396 | submit-korea@editage.com
