# Supplementary material for: Acupuncture Therapy for Military Veterans Suffering from Posttraumatic Stress Disorder and Related Symptoms: A Scoping Review of Clinical Studies
Source: Healthcare (Basel). 2023 Nov 14;11(22):2957. doi: 10.3390/healthcare11222957 (PMC10671227; doi:10.3390/healthcare11222957)
Supplement: Supplementary file 1 [file healthcare-11-02957-s001.zip › Table S1.pdf]

Table S1. Search terms used in each database

Medline via PubMed

	Searches	Results
#1	(“War Exposure[MeSH]” OR “Armed Conflicts” [MeSH] OR “Veterans”[MeSH] OR “Military Personnel“[MeSH] OR “Combat Disorders”[MeSH] OR War exposure[Title/abstract] OR Armed Conflicts[Title/abstract] OR Veterans[Title/abstract] OR Military Personnel[Title/abstract] OR war[Title/abstract] OR Combat[Title/abstract])	156,702
#2	("Acupuncture Therapy"[MeSH] OR "Acupuncture, Ear"[MeSH] OR "Acupuncture Points"[MeSH] OR "Acupuncture"[MeSH] OR "Electroacupuncture"[MeSH] OR "Meridians"[MeSH] OR acupuncture[Title/abstract] OR electroacupuncture[Title/abstract] OR electro-acupuncture[Title/abstract] OR acupoint*[Title/abstract])	35,301
#3	#1 AND #2	179

EMBASE via Elsevier

	Searches	Results
#1	war exposure/exp OR war exposure:ab,ti OR armed conflicts:ab.ti OR war/exp OR war:ab.ti OR Veterans/exp OR Veterans:ab.ti OR Military Personnel/exp OR Military Personnel:ab.ti OR Combat Disorders/exp OR Combat:ab.ti)	178,876
#2	(‘acupuncture’/exp OR ‘acupuncture’ OR ‘acupuncture therapy’ OR ‘auricular acupuncture’/exp OR ‘auricular acupuncture’ OR ‘ear acupuncture’ OR ‘acupuncture point’/exp OR ‘acupuncture point’ OR ‘electroacupuncture’/exp OR ‘electroacupuncture’ OR ‘electro-acupuncture’ OR ‘body meridian’/exp OR ‘body meridian’ OR ‘acupoint’)	62,049
#3	#1 AND #2	409

CENTRAL

	Searches	Results
#1	MeSH descriptor: [Armed Conflicts] explode all trees	155
#2	MeSH descriptor: [Veterans] explode all trees	1150
#3	MeSH descriptor: [Military Personnel] explode all trees	1039
#4	MeSH descriptor: [Combat Disorders] explode all trees	132
#5	MeSH descriptor: [War Exposure] explode all trees	22
#6	(armed conflicts OR veterans OR military personnel OR combat OR war OR war exposure):ti,ab,kw	9508
#7	#1 OR #2 OR #3 OR #4 OR #5 OR #6	9521
#8	MeSH descriptor: [Acupuncture] explode all trees	161
#9	MeSH descriptor: [Acupuncture Therapy] explode all trees	5208
#10	MeSH descriptor: [Acupuncture, Ear] explode all trees	213
#11	MeSH descriptor: [Electroacupuncture] explode all trees	875
#12	(Acupuncture OR Pharmacopuncture OR Ear acupuncture OR Pharmacoacupuncture OR Electroacupuncture OR Acupotomy OR Acupotomies OR Ear acupuncture OR Auricular acupuncture):ti,ab,kw	17184
#13	#8 OR #9 OR #10 OR #11 OR #12	17316
#14	#7 AND #13 in Trials	60

AMED via EBSCO

	Searches	Results
#1	(war exposure[TX] OR war[TX] OR armed conflicts[TX] OR veterans[TX] OR military personnel[TX] OR combat disorder[TX] OR combat[TX])	2556

#2	(“Acupuncture Therapy”[SU] OR “Acupuncture, Ear”[SU] OR “Acupuncture Points”[SU] OR Acupuncture[SU] OR Electroacupuncture[SU] OR Meridians[SU] OR acupuncture[TX] OR electroacupuncture[TX] OR electro-acupuncture[TX] OR acupoint*[TX])	12099
#3	#1 AND #2	31

CINAHL via EBSCO

	Searches	Results
#1	(MH “War+”) OR (MH “Veterans+”) OR (MH “Military personnel+”) OR (TX war exposure) OR (TX war) OR (TX armed conflicts) OR (TX veterans) OR (TX military personnel) OR (TX combat disorder) OR (TX combat)	122,841
#2	(MH “Acupuncture+”) OR (MH “Acupuncture, Ear+”) OR (MH “Acupuncture Points+”) OR (MH “Electroacupuncture”) OR (MH “Meridians+”) OR (TX acupuncture) OR (TX electroacupuncture) OR (TX electro-acupuncture) OR (TX acupoint*)	35,048
#3	#1 AND #2	**1176**

PsycARTICLES via ProQuest

	Searches	Results
#1	MAINSUBJECT.EXACT.EXPLODE("Military Personnel") OR MAINSUBJECT.EXACT.EXPLODE("Military Veterans") OR MAINSUBJECT.EXACT.EXPLODE("Combat Experience") OR war exposure OR war OR armed conflicts OR veterans OR military personnel OR combat disorder OR combat	
#2	MAINSUBJECT.EXACT.EXPLODE("Acupuncture") OR acupuncture OR acupuncture therapy OR (ear acupuncture) OR (acupuncture points) OR acupoint* OR electroacupuncture OR electro-acupuncture OR meridians	
#3	#1 AND #2	

OASIS

	Searches	Results
#1	(전쟁 OR 전투 OR 무력충돌 OR 군대 OR 군인) AND 침	**1**

KCI

	Searches	Results
#1	(전쟁 OR 전투 OR 무력충돌 OR 군대 OR 군인) AND 침	**32**

CNKI

	Searches	Results
#1	(SU='战争'+’战斗’+’武装冲突’ +’军队’+’军人’)AND (SU='acupuncture'+'针'+'鍼')	**99**

Wanfang data

	Searches	Results
#1	((((主题=战争) OR 主题=战斗) OR 主题=武装冲突) OR 主题=军队) OR 主题=军	**5178**

	人) AND (((主题=acupuncture) OR 主题=针) OR 主题=鍼)	

VIP

	Searches	Results
#1	M=(战争 OR 战斗 OR 武装冲突 OR 军队 OR 军人) AND M=(acupuncture OR 针 OR 鍼)	**42**

CiNii	Searches	Results
#1	(戦争 OR 戦闘 OR 武力衝突 OR 軍隊 OR 軍人) AND (acupuncture OR 針 OR 鍼)	**229**
