# Supplementary material for: Acupuncture Therapy for Military Veterans Suffering from Posttraumatic Stress Disorder and Related Symptoms: A Scoping Review of Clinical Studies
Source: Healthcare (Basel). 2023 Nov 14;11(22):2957. doi: 10.3390/healthcare11222957 (PMC10671227; doi:10.3390/healthcare11222957)
Supplement: Supplementary file 1 [file healthcare-11-02957-s001.zip › Table S2.pdf]

Table S2: List of Retrieved Full-Text Articles and Exclusion Reasons

Author		Title	inclusion	exclusion reason
Eisenlohr	2010	Acupuncture - A new option in the therapy of traumatized German soldiers?	O	
Hull	2012	Acupuncture and meditation for military veterans: Patient satisfaction and self reported symptom reduction	X	Inappropriate participant
Arhin	2016	Acupuncture as a Treatment Option in Treating Posttraumatic Stress Disorder밨elated Tinnitus in War Veterans	O	
King	2013	Acupuncture for sleep disturbances in veterans with post traumatic stress disorder	X	Duplicate
Niemtzow	2013	Acupuncture and its role in natural cataclysmic disasters and armed conflicts	X	Review article
Huang	2018	Acupuncture for treatment of persistent disturbed sleep: a randomized clinical trial in veterans with mild traumatic brain injury and post-traumatic stress disorder	O	
King	2015	Auricular acupuncture for sleep disturbance in veterans with post-traumatic stress disorder: a feasibility study	O	
Shao	2018	Battlefield Acupuncture (BFA) for pain management in a VA community living and rehabilitation center	X	Inappropriate participant
Chang	2010	AOM program helps vets recover from PTSD	X	Review article
Amini Rarani	2021	Effects of acupressure at the P6 and LI4 points on the anxiety level of soldiers in the Iranian military	X	Inappropriate intervention
Libretto	2014	Evaluation of an integrative PTSD treatment program	X	Inappropriate participant
Cronin	2013	Evaluation of the national acupuncture detoxification association protocol to treat combat stress induced insomnia	O	
King	2016	Exploring Self-Reported Benefits of Auricular Acupuncture Among Veterans With Posttraumatic Stress Disorder	O	
Prisco	2013	Group auricular acupuncture for PTSD-related insomnia in veterans: A randomized trial	O	
Conboy	2014	Management of gulf war syndrome symptoms with acupuncture: Findings of a wait-list controlled RCT	X	no outcome data
Vitzthum	2018	of post-traumatic stress disorder in soldiers and peacekeepers	X	Review article

Conboy	2009	Management of gulf war syndrome symptoms with acupuncture: Report on preliminary findings of an ongoing wait-list control RCT	X	Abstract
Engel	2014	Randomized effectiveness trial of a brief course of Acupuncture for posttraumatic stress disorder	O	
Schnyer	2012	The effectiveness of acupuncture in the treatment of Gulf War Illness	X	Inappropriate participant
Yurasek	2011	Acupuncture for PTSD in War Veterans	X	Review article
Fried	2012	Acupuncturists Without Borders: Disaster Relief; Community Clinics and Long-Term Trauma Recovery Through Acupuncture	X	Review article
Nct.	2009	Acuuncture for soldiers and veterans	X	Review article
Geib	2019	Acupuncture in veterans with permanent sleep disorders after mild traumatic brain injury	X	Inappropriate participant
Taylor-Swanson	2019	Matrix Analysis of Traditional Chinese Medicine Differential Diagnoses in Gulf War Illness	X	Inappropriate outcome
Smeeding	2010	Outcome evaluation of the Veterans Affairs Salt Lake City Integrative Health Clinic for chronic pain and stress-related depression, anxiety, and post-traumatic stress disorder	X	Inappropriate intervention
Silverstein	2015	Treating Our Veterans with PTSD	X	Review article
Chang	2009	Weighing the costs: integrative PTSD programs for veterans	X	Inappropriate outcome
趙名娟	2011	軍事應激下軍人心理狀態變化及中西醫結合干預?究	X	Inappropriate participant
趙名娟	2011	針灸聯合腦波治療儀干預軍人心理應激反應33例分析	X	Inappropriate participant
**赵璐**	2014	**针灸在中外军队的应用与研究**	X	Review article
权青云	2014	暗示及针灸治疗癔症性失语12例疗效观察	X	Inappropriate participant
钟周	2015	针刺联合团体性心理护理对优抚军人慢性疼痛伴发情绪障碍的效果观察	X	Inappropriate outcome
郭宏伟	2021	中医药拮抗创伤后应激障碍的研究现状	X	Review article
